# A novel compound heterozygous mutation in the *GJB2* gene causing non-syndromic hearing loss in a family

**DOI:** 10.3892/ijmm.2013.1581

**Published:** 2013-12-09

**Authors:** QINJUN WEI, YOUGUO LIU, SHUAI WANG, TINGTING LIU, YAJIE LU, GUANGQIAN XING, XIN CAO

**Affiliations:** 1 Department of Biotechnology, School of Basic Medical Sciences, Nanjing Medical University, Nanjing, Jiangsu 210029, P.R. China; 2 Department of Otolaryngology, The First Affiliated Hospital of Nanjing Medical University, Nanjing, Jiangsu 210029, P.R. China

**Keywords:** *GJB2*, hearing loss, prelingual, gene mutation

## Abstract

Mutations in the *GJB2* gene are responsible for up to 50% of cases of non-syndromic recessive hearing loss, with c.35delG, c.167delT and c.235delC being the predominant mutations in many world populations. However, a large number of rare mutations in this gene may also contribute to hearing loss. The aim of the present study was to conduct a clinical and molecular characterization of a Chinese family with non-syndromic hearing loss. Sequence analysis of the *GJB2* gene led to the identification of a novel compound heterozygous mutation c.257C>G (p.T86R)/c.605ins46 in two profoundly deaf siblings whose hearing parents were each heterozygous, either for the c.257C>G (paternal) or for the c.605ins46 (maternal) mutations. Both c.257C>G and c.605ins46 are rare *GJB2* mutations that have previously been reported to segregate with autosomal recessive hearing loss exclusively in East Asian populations. To study the pathogenic effect of the compound heterozygous mutation, a three-dimensional model was constructed and Anolea mean force potential energy was predicted for a bioinformatic structural analysis. HEK293 cells were used to study the pathogenic effect of mutant connexin 26 proteins. The results suggested that the c.257C>G (p.T86R)/c.605ins46 mutations in the *GJB2* gene provides a novel molecular explanation for the role of the *GJB2* gene in hearing loss.

## Introduction

Hearing loss is one of the most common sensory disorders in humans. Approximately half of hearing loss cases have a genetic etiology with autosomal dominant, autosomal recessive, X-linked, or mitochondrial modes of inheritance (1), with autosomal recessive being the most common. There are two monogenic forms of hearing loss including syndromic or non-syndromic ones. Approximately 71 genes have been described for non-syndromic hearing loss (as mentioned in the hereditary hearing loss homepage, http://hereditaryhearingloss.org/). The gene most frequently involved in autosomal recessive non-syndromic hearing loss is *GJB2*, which encodes the gap junction protein connexin 26 (Cx26) and is responsible for over half of the cases, followed by *SLC26A4*, *MYO15A*, *OTOF*, *CDH23* and *TMC1* (2). Over 150 mutations, polymorphisms and unclassified variants have been identified in the *GJB2* gene (http://davinci.crg.es/deafness), some of which are frequent, while others are extremely rare. These mutations occur at different frequencies across populations (3), with c.35delG, c.167delT and c.235delC predominating in Caucasian, Ashkenazi Jewish and East Asian populations, respectively (4–8). In addition, Pendred syndrome mutations in *SLC26A4* account for 10% of hereditary hearing loss in most world populations. In China, almost 50% of patients with nonsyndromic hearing loss carry the *GJB2* or *SLC26A4* mutations (8). Identification of these mutations is of primary interest in genetic counseling. Although a large number of cases are caused by hotspot mutations of these genes as revealed by molecular epidemiologic studies, rare mutations may also contribute to hearing loss.

In this study, we reported the identification of a novel compound heterozygote with two missense mutations in the *GJB2* gene, and assessed the pathogenic effects of these mutations based on bioinformatic structural analysis and the subcellular localization of the compound heterozygous mutant Cx26 protein in HEK293 cells.

## Materials and methods

### Subjects and clinical examinations

**Figure 1:**
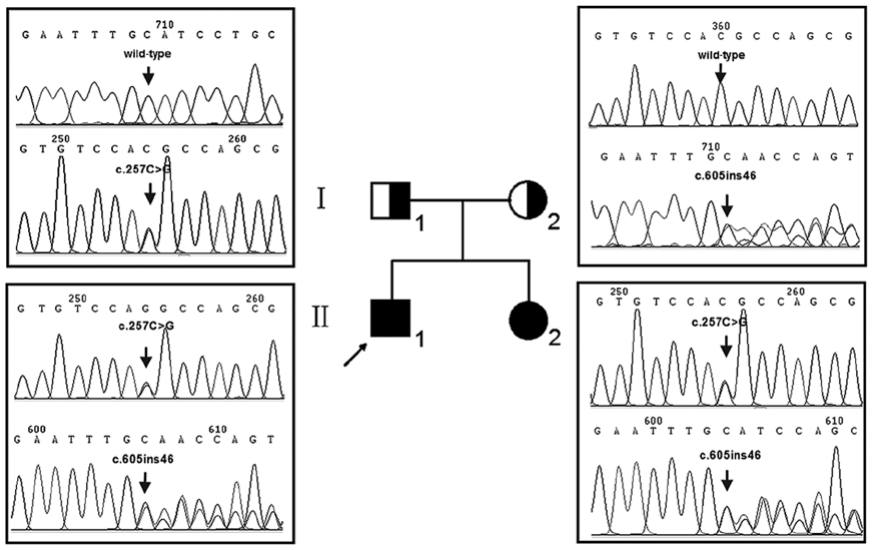
Pedigree and genotypes of the family showing the novel compound heterozygous *GJB2* c.257C>G (p.T86R) and c.605ins46 mutations.

Two siblings (II-1 and II-2) (Fig. 1) of Chinese Han origin suffering from prelingual hearing loss were referred to our departments for clinical and molecular evaluation. Informed consent was obtained from their parents prior to their participation in the study, which was conducted in accordance with the Ethics Committee of the First Affiliated Hospital of Nanjing Medical University. A comprehensive history and physical examination were performed to identify any syndromic findings, the history of the use of aminoglycosides, and genetic factors related to hearing loss. Audiological studies including pure tone audiometry, auditory brainstem response (ABR), immittance and distortion product otoacoustic emissions (DPOAEs) were conducted in a soundproof room. The pure-tone average was calculated from the sum of the audiometric thresholds at 500, 1,000 and 2,000 Hz. The severity of hearing loss was classified into five grades: normal, <26 decibel (dB); mild, 26–40 dB; moderate, 41–70 dB; severe, 71–90 dB; and profound, >90 dB.

### Molecular analysis

Genomic DNA was isolated from EDTA-anticoagulated blood samples of the two siblings and their parents using Puregene DNA Isolation kits (Gentra Systems, Minneapolis, MN, USA). Nine hotspot mutations of deafness genes present in Chinese populations were screened by using a universal array approach, termed a multiplex allele-specific PCR-based universal array (ASPUA), as previously described (9). The mutations included c.35delG, c.176del16bp, c.235delC and c.299delAT in the *GJB2* gene, c.538C>T in the *GJB3* gene, c.IVS7-2A>G and c. 2168A>G in the *SLC26A4* gene, and m.1555A>G and m.1494C>T in the *RNR1* gene of mitochondrial DNA (mtDNA). The participants were then subjected to bidirectional sequencing of the coding region of the *GJB2* gene to investigate the existence of possible rare or novel pathogenic mutations (methods are available upon request). Samples from 400 unrelated Chinese individuals with normal hearing were collected served as controls.

### Computer-assisted model building and structure-based analysis

3D models of the human wild-type (WT) and mutant Cx26 proteins were constructed using SWISS-MODEL (Basel, Switzerland) (10–12). The SWISS-MODEL (http://swissmodel.expasy.org/) is a server for the automated modeling of 3D protein structures, and the resulting protein can be visualized and analyzed using visual molecular dynamics (VMD) 1.9 (http://www.ks.uiuc.edu/Research/vmd/vmd-1.9/). By comparing the 3D protein structures and Anolea mean force potential energy of the WT and mutant Cx26 proteins, we evaluated the effect of *GJB2* mutations on the protein structure (13).

### Molecular cloning of WT and mutant GJB2 genes

A WT human *GJB2* sequence fragment cDNA was subcloned into the pEGFP-N1 and pmCherry-N1 vectors to construct Cx26-EGFP and Cx26-mCherry fusion proteins. The mutant *GJB2* sequences were obtained from the genomic DNA of the proband carrying the compound heterozygous mutation (c.257C>G/WT, c.605ins46/WT). PCR was conducted using the primers that contained *Hin*dIII and *Bam*HI restriction sites: forward, 5′-GCGCAAGCTTTATGGATTGGGGCACGCT-3′ and reverse, 5′-GCGCGGATCCCTAACTGGCTTTTTTGAC-3′. The sequences of Cx26-c.257C>G and Cx26-c.605ins46 were confirmed by DNA sequencing.

### Immunocytochemical analysis of Cx26 WT and mutant in HEK293 cells

The HEK293 cell line, which is deficient for gap junctions, was obtained from the American Type Culture Collection (ATCC, Manassas, VA, USA). HEK293 cells were grown in the recommended medium consisting of Dulbecco’s modified Eagle’s medium, 10% fetal bovine serum (both from Invitrogen, Carlsbad, CA, USA), and 0.5% penicillin and streptomycin. The plasmids encoding WT or mutated Cx26 tagged with fluorescent protein markers were transfected to the cells using X-tremeGENE HP transfection reagent (Roche Diagnostics, Indianapolis, IN, USA) according to the manufacturer’s instructions. The intracellular localization of WT and mutant Cx26 proteins was investigated by immunocytochemical analysis 48 h after transfection.

## Results

### Clinical and molecular analysis

The two children suffered from severe to profound congenital hearing loss that was confirmed by consecutive previous ABR tests. The patients presented to the Department of Otolaryngology at the First Affiliated Hospital of Nanjing Medical University at the ages of 13 and 16 years. Complete medical histories showed that neither child had any abnormal history during pregnancy or delivery, or had a history of exposure to aminoglycosides prior to their deafness. Physical and otoscopic examination failed to identify any syndromic findings. Unaided audiometric testing indicated bilaterally symmetric and profound sensorineural hearing loss in each of the two siblings, with normal results for their parents. Other audiological examinations, including immittance, ABR and DPOAEs, revealed cochlear involvement.

Following molecular screening by ASPUA, hotspot mutations in *GJB2*, *SLC26A4*, *GJB3* and mtDNA *RNR1* genes were excluded as causative factors of the hearing loss of the two children. Subsequent sequence analysis of the *GJB2* gene, however, revealed that both the profoundly deaf children were compound heterozygotes for a previously unreported combination of the *GJB2* mutations, c.257C>G (p.T86R) and c.605ins46 (Fig. 1). The parents were each unaffected heterozygotes, the father a c.257C>G heterozygote and the mother a c.605ins46 heterozygote. The c.257C>G and c.605ins46 mutations were not detected in 400 Chinese controls.

### Comparison of 3D structure and Anolea mean force potential of WT and mutant Cx26 proteins

To understand the mechanism of the mutations at a protein level, 3D models of WT and mutant Cx26 proteins were constructed for bioinformatic structural analysis. The results obtained by SWISS-MODEL demonstrated that the c.257C>G mutation is located in transmembrane domain 2 (TM2) and the c.605ins46 mutation is in the TM4 of Cx26. These mutations may change the Cx26 structure both in transmembrane domains and in the extracellular loop and may play a significant role in the development of hearing loss.

**Figure 2:**
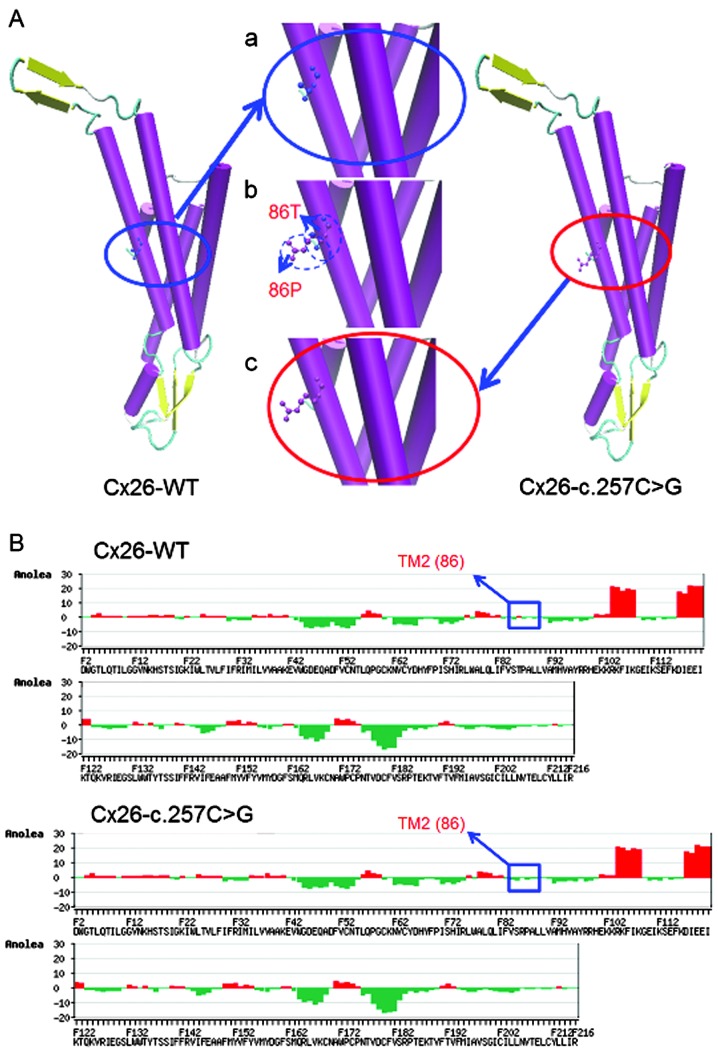
Bioinformatic estimation of the 3D structures of the wild-type (WT) and c.257C>G Cx26 proteins. The secondary structure of Cx26 is shown by solid purple cylinders (α-helices), yellow arrows (β-sheet) and white loops (turn). (A). 3D structures of the Cx26-WT and Cx26-c.257C>G proteins (blue, amino acid residue position before mutation; red, position after mutation). Local 3D structures of (a) the WT protein, (c) the Cx26-c.257C>G protein and (b) their overlapping structures are shown (blue and red indicate amino acid residues of the WT and Cx26-c.257C>G proteins, respectively). (B). The graphs show computer modeling from the SWISS-MODEL workspace. An evaluation was performed using the Anolea mean force potential to assess the quality of the model and the stability of the folding of protein chains. The Anolea mean force potential changed the WT (top) and c.257C>G (bottom) versions of the Cx26 protein. The arrows and boxes indicate amino acid residue 86, located in the TM2 region of the human Cx26 protein.

**Figure 3:**
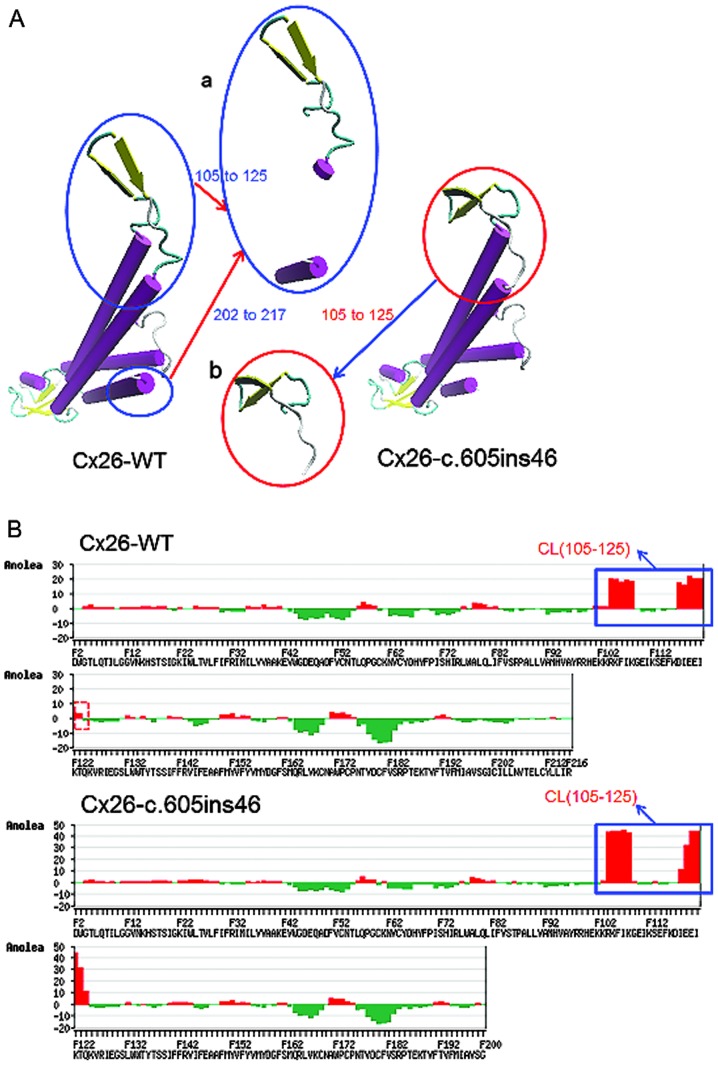
Bioinformatic estimation of the 3D structures of the wild-type (WT) and c.605ins46 Cx26 proteins. (A). 3D structures of the Cx26-WT and Cx26-c.605ins46 proteins (blue, amino acid residue position before mutation; red, position after mutation). The local 3D structures of the (a) WT and (b) Cx26-c.605ins46 proteins are shown. (B) The graphs show computer modeling from the SWISS-MODEL workspace. An evaluation was performed using the Anolea mean force potential to assess the quality of the model and the stability of the folding of protein chains. Anolea mean force potential changes of the WT (top) and c.605ins46 (bottom) versions of the Cx26 protein. The arrows and boxes indicate amino acid residues 105–125, located in the cytoplastic loop (CL) region, and residues 202–217, located in the TM4 region of the human Cx26 protein.

Overlapping analysis of the 3D structures of the Cx26-WT and Cx26-c.257C>G proteins revealed distinct changes at residue 86 (Fig. 2A). Anolea mean force potential energy analysis revealed one region with marked changes (Fig. 2B). The significant increase of atomic potential energy in this region may change the Cx26 structure from a stable low-energy state to an unstable higher-energy state. Overlapping analysis of the Cx26 structure with the c.605ins46 mutant (Fig. 3A) also showed distinct changes of spatial structure at residues 105–125 and 202–215, located in the cytoplastic loop (CL) and TM4, respectively. Anolea mean force potential energy analysis revealed two regions with marked changes (Fig. 3B). As a result, the c.257C>G and c.605ins46 mutations are capable of altering the spatial structures of some amino acid residues in the transmembrane domain, thereby preventing the effective functioning of Cx26.

### Cx26 protein expression and subcellular localization in HEK293 cells

**Figure 4:**
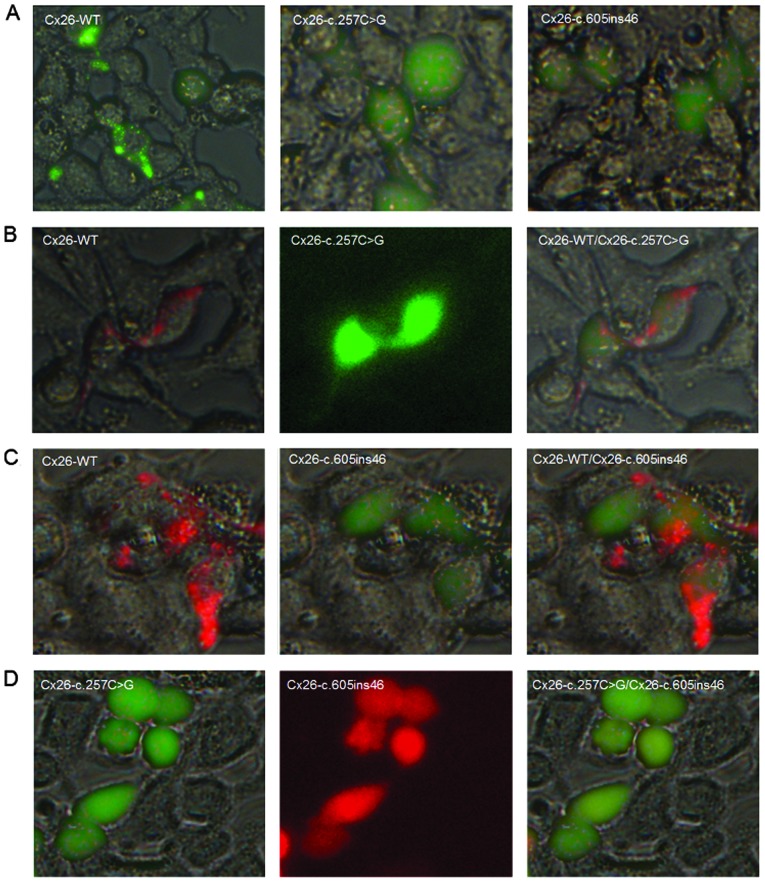
Immunocytochemical analysis of wild-type (WT) and mutant Cx26 protein expression in transfected HEK293 cells. (A). The cellular localization of wild-type and mutant Cx26 proteins. The Cx26-WT protein localized to the membrane and formed gap junction plaques between adjacent cells, unlike the mutant Cx26 proteins. (B). Co-expression of Cx26-WT-mCherry and Cx26-c.257C>G-EGFP. The gap junction is visible between cell pairs. (C) mCherry-tagged Cx26-WT co-expressed with EGFP-tagged Cx26-605ins46 also formed gap junctions in cell pairs. (D) Co-expressed Cx26-c.257C>G-EGFP and Cx26-605ins46-mCherry did not form gap junctions but were expressed in the cytoplasm.

To examine the effects of the c.257C>G and c.605ins46 mutations on the cellular localization of Cx26, we transfected expression plasmids encoding WT or mutant Cx26 into HEK293 cells. The proteins were fluorescently tagged to track their intracellular locations. Cx26-WT localized at the cell membrane and formed gap junctions, as indicated by the characteristic plaques between two adjacent cells (Fig. 4A). By contrast, the Cx26-c.257C>G and Cx26-c.605ins46 mutants were not expressed at the cell membrane and lacked the ability to form gap junctions. When the mCherry-tagged Cx26-WT protein was co-transfected with the Cx26-c.257C>G mutant, the mutant protein did not interact with Cx26-WT to co-assemble into the same gap junction plaques, however, gap junction plaques were still evident at the cell-cell contact areas (Fig. 4B). The Cx26-c.605ins46 mutant behaved in a similar manner (Fig. 4C). However, the Cx26-c.257C>G protein was unable to interact with the Cx26-c.605ins46 mutant to co-assemble into the same gap junction plaques (Fig. 4D). Therefore, we suggest that the mechanism of hearing loss caused by the c.257C>G/Cx26-c.605ins46 mutation is due to the inability of the mutant Cx26 proteins to be transported to the cell membrane.

## Discussion

In the current study, we present a novel compound heterozygous mutation in the *GJB2* gene, the c.257C>G/c.605ins46 mutation, which resulted in non-syndromic sensorineural hearing loss in a Chinese family. Both of the affected siblings suffered from prelingual hearing loss, while their father and mother (who were each heterozygous for one of the individual mutations) exhibited normal hearing function, suggesting an autosomal recessive pattern of inheritance in this family. This information may advance our understanding of the pathogenic mechanism of *GJB2* mutations associated with hearing loss.

c.257C>G and c.605ins46 are rare *GJB2* mutations that have previously been reported to segregate with hearing loss exclusively in East Asian populations, either homozygously (8,14–16) or as part of compound heterozygous mutations with other more prevalent mutations such as c.235delC and c.299delAT (8). It is theoretically a very improbable event for these two rare mutations to occur in one patient. To the best of our knowledge, this is the first international study to demonstrate that the compound heterozygous mutations c.257C>G and c.605ins46 are associated with non-syndromic recessive hearing loss in a family. Results of this study demonstrate the limitations of screening as only the most prevalent mutations of the *GJB2* gene were used to identify causative factors in hearing loss patients. Therefore, it is preferable to sequence the entire coding sequence of this gene when a genetic cause cannot be excluded.

*GJB2* mutations have been shown to cause variable hearing loss phenotypes even among family members (17,18). However, the c.257C>G/c.605ins46 compound heterozygous state led to profound deafness in the two affected siblings in the studied family. The c.257C>G mutation, which is located in the second transmembrane domain of Cx26, converts an uncharged amino acid (threonine) at codon 86 to a positively charged amino acid (arginine) and produces a functionally null protein (14). It is believed that the hearing loss caused by this mutation stems from its inability to localize to the cell membrane (16). c.605ins46, a frame-shift mutation, is sited in the fourth transmembrane domain of Cx26 and has a tandem repeat of 46 nucleotides at position 605. A stop codon (TGA) is introduced at the 202nd amino acid, leading to premature termination of polypeptide synthesis. To clarify the function of these proteins and the pathological mechanisms of the mutations, we constructed a 3D characterization of protein structures (13,19). Based on 3D modeling and Anolea mean force potential energy analysis (10–13,20), we investigated the molecular mechanisms of the c.257C>G/c.605ins46 compound heterozygous mutation in the *GJB2* gene by analyzing the protein structure of Cx26. The c.257C>G/c.605ins46 mutation caused changes in the spatial structure of neighboring amino acids, preventing Cx26 from functioning efficiently (21–23). In the current study, protein localization and gap junction function were investigated by transfecting fluorescently tagged WT or mutant Cx26 into HEK293 cells, which allowed visual confirmation of homozygous or heterozygous mutant gap junctions. When the Cx26-c.605ins46 or the Cx26-c.257C>G mutant was transfected together with the Cx26-WT protein, gap junction plaques were observed at cell-cell contact areas, although the mutant proteins were unable to interact with Cx26-WT to co-assemble into the same gap junction plaques. However, the Cx26-c.257C>G mutant was unable to interact with the Cx26-c.605ins46 mutant to co-assemble into gap junction plaques. A similar biological effect would be expected if the above event occurred in the hair cells of the inner ear, in other words, that the individual’s normal hearing function would be affected.

In conclusion, to the best of our knowledge, this is the first description of the compound c.257C>G/c.605ins46 mutation of *GJB2* in a Chinese family with non-syndromic hearing loss. The c.605ins46 mutation causes a frame-shift and premature termination at amino acid 202, while c.257C>G is a missense mutation converting codon 86 from threonine (T) to arginine (R). Further bioinformatic structural analysis and cell-based functional assays indicated the pathogenic mechanism of this compound heterozygous mutation in the *GJB2* gene associated with hearing loss. This information is valuable for understanding the pathogenic roles of Cx26 mutations associated with hearing loss and for determining their genetic diagnosis.
